# The British Journals

**Published:** 1841-04

**Authors:** 


### II. THE BRITISH JOURNALS.

(FOR THE QUARTER ENDING MARCH 1841.)

---

*List of the principal Medical Papers published in the British Journals during last Quarter.*

### THE EDINBURGH MEDICAL AND SURGICAL JOURNAL.

No. CXLVI. JAN.—On the Anatomical Relations of the Blood-vessels of the Mother to those of the Fœtus in the Human Species. By John Reid, M.D.

On some points in the Anatomy of the Medulla Oblongata. By John Reid, M.D.

The Properties and Influence of Arteries on the Circulation of Blood. By G. Calvert Holland, M.D.

An Inquiry into the Mechanical Functions of the Ear. By James Sym, M.D.

Case of Aortal Aneurism, which opened into the Vena Cava Superior, opposite the entrance of the Vena Azygos. By William Young, M.D.

Two Cases of Impracticable Labour arising from Malcosteon of the Pelvis, in which the Cæsarean Operation was performed; accompanied with Practical Observations. By Thomas Radford, M.D.

A Description of a Double Monocephalic Human Monster, which was transmitted to this country from South America, by Robert Mackay, Esq., British Consul at Maracaylis, Venezuela. By Eric Mackay, M.D.

Malta, considered with reference to its eligibility as a Place of Residence for Invalids. By Francis Sankey, M.D.

On the Diurnal Variations of the Pulse in Disease. By William Augustus Guy, M.B. Cantab.

Observations on the Use of Piperine in the Treatment of Intermittent Fever. By Robert Hartle, M.D.

[In cases where quinine failed, Dr. Hartle found (in Trinidad,) the piperine succeed, in doses of 3 grs. every hour.]

Observations on the Therapeutic Action of Croton Oil in certain Nervous Disorders. By P. S. K. Newbigging, M.D.

Case of Amputation of the Neck of the Womb followed by Pregnancy ; with Remarks on the Pathology and Radical Treatment of the Cauliflower Excrescence from the Os Uteri. By James Y. Simpson, M.D.

On Sanguineous Tumours on the Scalp of New-born Children. By Francis Black, M.D.

Two Cases of Rupture of the Carotid Artery from Sphacelus. By Mr. C. J. Mill.

[In cynanche maligna.]

Cases Illustrative of the Division of Tendons. By Mr. W. Rhind, S urgeon.

Appendix to Dr. J. Reid's Paper on the Anatomical Relations of the Blood-vessels of the Mother to those of the Fœtus.

---

### EDINBURGH MONTHLY JOURNAL OF MEDICAL SCIENCE.

No. I. JAN.—Surgical Cases and Observations. By James Syme, Esq. F.R.S.E. &c.

On the Pathology of Laryngismus Stridulus. By W. Henderson, M.D. Edinburgh, &c.

Contributions to Forensic Medicine. By John Reid, M.D. Edinburgh, &c.

Case of Scirrhus of the Stomach, probably Congenital, with Remarks. By Thomas Williamson, M.D. Edinburgh, &c.

Case of Luxation during a Fit of Epilepsy, with Remarks. By W. A. F. Browne, M.D. &c.

On the Preparation of Protiodide of Iron. By T. and H. Smith, Chemists.

On the Cause of Ciliary Motion. By Edward Forbes, M.W.S. &c.

No. II. FEB.—On the Varieties observed in the Symptoms, &c. of Poisoning with Opium. By David Skae, Esq. F.R.C.S. Lecturer on Forensic Medicine, &c.

On Bleeding in Mania. By W. A. F. Browne, M.D. Superintendent of the Crichton Lunatic Asylum, Dumfries.

On the Changes of the Colour of the Iris, produced by Inflammation. By James Hunter, M.D. Surgeon to the Edinburgh Eye Dispensary.

Remarkable Case of Aneurism of the Aorta, with Remarks. By David Ure, Esq. Surgeon, Dundee.
Case of Fibrous Tumour surrounding the Right Sciatic Nerve. By James Duncan, M.D. F.R.C.S.E. one of the Surgeons of the Royal Infirmary of Edinburgh.
On the Employment of Opium for arresting Acute Internal Inflammations. By Robert Christison, M.D. F.R.S.E. Professor of Materia Medica in the University of Edinburgh.
Case of Division of the Tendo-Achillis, with Remarks. By James Miller, F.R.C.S.E. one of the Surgeons of the Royal Infirmary of Edinburgh, Lecturer on Surgery, &c.

No. III. MARCH.—Cases in Surgery from the Clinical Practice of Professor Syme. Reported by Dr. Hardie.
On the Sources of Hemorrhage after Lithotomy. By James Spence, Esq. Surgeon. With Lithographic Illustrations.
On the Operations for Strabismus, Action of the Oblique Muscles, &c. By D. Ross Lietch, M.D. Edinburgh.
Cases and Observations illustrative of the Effects of the Contraction of Cicatrices and False Membranes. By John Rose Cormack, M.D. Edinburgh, Fellow of the Royal College of Physicians, and Physician to the Royal Dispensary of Edinburgh.
Pathological Illustrations of Sudden Death. By J. R. W. Vose, M.D. Senior Physician to the Northern Dispensary of Liverpool.

---

### THE DUBLIN JOURNAL OF MEDICAL SCIENCE.

No. LIV. JAN.—A Case of Tumour of the Brain, with Hydrocephalus, and Arrest of Development of the Uterine System. By John O'Bryen, M.D. &c. Clifton.
On the Development of the Embryo in the different Classes of the Animal Kingdom. By John Scouler, M.D. Professor of Geology in the Royal Dublin Society.
Researches in Operative Midwifery. By Fleetwood Churchill, M.D. No. V.—On the Perforator and Crotchet.

No. LV. MARCH.—Practical Observations on the Diagnosis and Treatment of some functional Derangements of the Heart. By D. J. Corrigan, M.D. Dublin.
An Account of an Epidemic Ophthalmia which prevailed among the Children of the South Union Workhouse during the Summer and Autumn of 1840. By Cathcart Lees, M.B. Physician to the Institution, and to the Pitt-street Institution for the Diseases of Children.
Observations on a Mode of introducing the Catheter in difficult Cases. By Charles Patterson, M.D. Physician to the Rathkeale Infirmary and Fever Hospital.

[This plan, taken from a method recommended by Dr. O'Beirne in cases where difficulty is experienced in introducing the elastic tube into the intestinal canal, consists in injecting warm water through the catheter while it is being gently forced past the obstruction.]

"The injecting apparatus which I have employed is very simple; it is formed by merely attaching a small bladder to the end of the catheter, after the manner of a mounted enema pipe; the bladder need not be large, one that holds about half a pint of water will be sufficiently so; and the catheter must be furnished with a small cork, having a piece of twine fixed to it, just like the cork of an enema pipe. The largest sized catheter the urethra will admit of should be used, that the impetus of as large a jet of water as possible may act on the obstructed part of the canal, and also that the urethra being filled by the instrument, the fluid may be easily prevented from returning by the side of the catheter. The eye must be large, and very near the end of the tube; or, what is better, the tube should have a large orifice within the circumference of its anterior extremity, with the edge rounded in so as that it shall not hurt or catch the lining membrane of the passage. Also it must be a silver or permanently curved elastic catheter: for the injecting apparatus would prevent the withdrawal of the stilet necessary to maintain the curvature of a common elastic one. The instrument, having the attached bladder properly adjusted, is to be passed down to the obstruction: if it cannot now, on further trial, be made to enter the patient's bladder, it must be held by an assistant, while the small cork is being inserted into its outer end: about six ounces of lukewarm water are to be poured into the injecting apparatus; the latter must then be closed, and tied as near as possible to the fluid, so as to exclude every portion of air. The operator is next to encircle the penis with the finger and thumb of his left hand, making gentle circular pressure to close the urethra round the catheter; he will then, having first withdrawn the cork, embrace the bladder of water with his right hand, so as to be able to apply strong and uniform pressure on as much of its surface as possible, in doing which, its contents being continuously and forcibly propelled into the urethra, and the handle of the instrument being at the same time depressed, the latter at once passes with facility into the patient's bladder."

[This method has been successfully employed by Dr. Patterson in some cases of enlarged prostate, and also in one case where the difficulty originated in inflammation of the urethra.]

On Asphyxia, resulting from Wounds in the Neck. By Gabriel Stokes, M.D.

Cases of the Venereal Disease, illustrative of the Introduction of the Venereal Poison into the System, through other Channels than by Sexual Intercourse. By Clement Hamerton, Surgeon to the Castletown Dispensary, Nobber.

An Attempt to estimate some of the characteristic Marks, by which to judge of the Cause of Perforations of the Stomach. By Thomas Williamson, M.D. one of the Physicians to the Leith Dispensary, &c.

Clinical Report of Sir Patrick Dun's Hospital. By Charles Lendrick, M.D. T.C.D. Queen's Professor of the Practice of Medicine, &c.

---

### THE LANCET.

No. XI. Dec. 5.—Observations on the Effects of Dividing the Tendons of the various Orbital Muscles in Animals. By E. W. Duffin, Esq. Surgeon, London.

On the Proper Mode of Treating the Insane. By Arthur Stilwell, Esq. Surgeon.

[Advocates mild restraint in certain cases.]

On the Tincture of the Muriate of Iron in Discharges of Blood from the Urethra, Leucorrhœa, and Dysmenorrhœa. By Charles Clay, Esq. Surgeon, Manchester.

[Says he has cured many cases by the tinct. ferr. ♍ viij. and tinct. opii ♍ xj. every four hours.]

On the Application of Strapping and Pressure in Orchitis. By J. Dixon, Esq. Surgeon, Coleford.

[Fricke's method. (See Br. and For. Med. Rev. vol. II. p. 253.) Mr. D. wrongly gives the merit of the plan to Mr. Cross.]

No. XII. Dec. 12.—Injection of Nitrate of Silver in Gonorrhœa. By W. H. Foster, Esq.

[Recommends an injection of a solution of nitrate of silver, gr. iij. to the oz. in the primary stage of gonorrhœa.]

Medical Jurisprudence. Inquests in Middlesex, held before Mr. Wakley, M.P. Recorded by Mr. George I. Mills. Sudden Deaths—Proximate Causes.

Hemorrhagic Diathesis: Cases in Illustration. By David Burnes, M.D. London.

[Good examples of this not very uncommon diathesis in three brothers: one case fatal, with the dissection.]

Retained Placenta after Premature Labour. By Charles Ray, Esq. Surgeon, London.

[Single case. Placenta retained thirty-one days.]

Additional Observations on Sulphur Lotum in Rheumatic Affections, and the Operation for Varicose Veins in the Legs. By Charles Clay, Esq. Surgeon, Manchester.

Case of Stricture of the Œsophagus. By Charles Gravenor, Esq. London.

No. XIII. Dec. 19.—Medical Jurisprudence. Inquests in Middlesex, held before Mr. Wakley, M.P. Recorded by Mr. George I. Mills.

On the Humane System of Treating the Insane. By Andrew Blake, M.D. Physician to am General Lunatic Asylum.

Effects of Restraint on the Insane. By Arthur Stilwell, Esq. Surgeon.

No. XIV. Dec. 26.—Medical Jurisprudence. Inquests in Middlesex, held before Mr. Wakley, M.P.

Operation for Lateral Curvature of the Spine. By G. B. Childs, Esq. London.

[Single case.]

Case of Epileptic Catalepsy. By James Thomas, Esq. Newcastle Emlyn.

No. XV. Jan. 2.—Case of Strangulated Femoral Hernia; Operation; Successful Result. By Charles Braddon, Esq. Surgeon, Crediton.

Case of Hydatids in the Brain, unattended with Symptoms during Life. By William Sturton, Esq. Surgeon, Greenwich.

On the Injurious Employment of Emmenagogues in Vicarious Discharges. By Edward Boulger, Esq. Surgeon, Reading.

[Single case.]

Objections to the Use of Nitrate of Silver as an Internal Remedy. By Charles Ray, Esq. Surgeon, Eaton square.

On the newly-discovered and speedily-forgotten Instruments, and the new Plans and the old Plans, for performing the simple Operation for the Cure of Squinting. By Charles Clay, Esq. Surgeon, Manchester.
On the frequent Occurrence of Caries in the Temporal Bones. By William Hughes, Esq. Surgeon, Holborn.
On the Non-existence of Infection in Gonorrhœa. By Mr. Chippendale.
On the Erroneous Supposition that a Cicatrix after Vaccination is Proof of a Successful Operation. By John Epps, M.D.
On the Advantages of the Sling Fracture-bed in Cases of Fracture, with Illustrative Cases. By H. G. Potter, F.L.S. Surgeon, Newcastle-upon-Tyne.

No. XVI. JAN. 9.—On the Humane System of Treating the Insane. By Andrew Blake, M.D. Physician to the Nottingham General Lunatic Asylum.
On the Treatment of the Insane in Private Houses. By George Stilwell, Esq.
Statistics of Multiple Births. By R. H. Mackenzie, M.D. M.R.C.S. Chelsea.
Case of Retained Placenta and Polypus of the Uterus. By P. L. Burchell, Esq. Surgeon.
On the Poisonous Effects of Laburnum Seeds. By T. A. B. Bonney, Esq. Surgeon.
Double Uvula; from Median Arrest of Development. By George Bolster, Esq. Surgeon, London.

No. XVII. JAN. 16.—On Acute Rheumatism; its Causes, Symptoms, and Mode of Treatment. By David D. Davis, M.D. Professor of Midwifery in UniversityColl. London.
On the Action of Sulphuric Acid on the Cornea. By R. Dundas Thomson, M.D. London.
Collection of Fluid in the Substance of the Right Lobe of the Thyroid Body. By J. Massey, Esq. Surgeon, Nottingham.

No. XVIII. JAN. 23.—Cases of Aneurism of the Thoracic Aorta. By Robert Boyd, M.D. Resident Physician to the St. Marylebone Infirmary, &c.
Case of Suspected Empyema. By J. Gorringe, Esq. University College Hospital.
On the Employment of Oxymuriatic Acid as a Remedy for Disease. By F. d'Alquen, Esq.

No. XIX. JAN. 30.—On the Minute Pathology of Pneumonia. By Thomas Williams, M.B. Surgeon, Demonstrator of Anatomy in the Webb-street School of Medicine.
A New and Successful Method of Treating Disease of the Prostate Gland; with a Wood Engraving. By W. Henderson, M.D. Perth.

[The new method consists in the application of leeches near the affected part, within the rectum, by means of a particular tube.]

On the Treatment of the Insane. By Arthur Stilwell, Esq. Surgeon.
Cases Illustrative of Hemorrhagic Diathesis. By T. Smethurst, Esq. Surgeon, Ramsgate.

[Two cases (females) not of the same family.]

No. XX. FEB. 6.—Reflections on the Treatment of Variola by the "Ectrotic Method;" principally with a View of Preventing the Formation of Cicatrices. By J. F. Olliffe, M.D. President of the Parisian Medical Society.

[An elaborate essay on the method of treating smallpox by the local application of mercurial plasters, noticed more than once in this Journal. Dr. Olliffe speaks most favorably of the new plan. "Its effect is either to prevent the development of the pustules, or so to modify them that they become mere abortions."]

Case of Aneurism of the Pulmonary Artery. By S. W. Fearn, Esq. Surgeon, Derby.
On the Substitution of the Oxide of Siiver for the Nitrate. By Thomas P. Dennett, Esq. Surgeon, Storrington.

[In the treatment of epilepsy, &c. with the view of avoiding the cutaneous discoloration.

On the Humane System of Treating the Insane; Letters from Dr. Blake, of Nottingham, and Dr. Browne, Physician to the Crichton Institution.
On the Employment of Brandv and Salt. By Robert Dick, M.D.
On the Treatment of Sciatica. By H. C. Roods, Esq. Surgeon, London.

No. XXI. FEB. 13.—On the History of the Employment of Cinchona Bark in the Treatment of Acute Rheumatism. By David D. Davis, M.D. Professor of Midwifery in University College.
On Ivory Exostosis of the Fibula; Removal of Eight Inches and a Half in Length of the Diseased Bone. By J. Massey, Esq. Surgeon, Nottingham.
On a Peculiar Form of Infantile Convulsions. By W. J. West, Esq. Surgeon, Tunbridge.
Epistaxis; Cured by Plugging the Nares with Putty.
On the Employment of the Unguentum Æruginis in the Treatment of Burns and Scalds. By Edwyn G. S. Gurney, Esq. Surgeon, Camborne.

Removal of a Pendulous Tumour from one of the Labia Pudendi. By William Howitt, Esq. Senior Surgeon of the Preston Dispensary.

No. XXII. Feb. 20.—An Outline of Chinese Anatomy. With illustrative Wood Engravings. By G. Tradescant Lay, Esq. Surgeon, London.
On a Case of supposed Aneurism of the Aorta, relieved by appropriate Treatment. By John Gay, Esq. Surgeon, London.
On the Employment of Ergot of Rye in Premature Labour. By Joseph Hodgson, Esq. Surgeon, London.
[A single case.]

No. XXIII. Feb. 27.—New Method of Treating Cases of Purely Functional Neuralgy. Exhibition of the Galvanic Factors. By Robert Dick, M.D.
[This method consists in "the simultaneous administration (internally) of the ordinary *galvanic factors,* zinc, copper, or nitric acid."]
On the Application of the Sling Fracture Bed to the Treatment of Compound Fractures of the Leg. By William Morrison, Esq. Durham.
On the Employment of Burnt Rhubarb in Diarrhœa. By F. P. Hoblyn, Esq. London.
[Strongly recommended. The rhubarb is burnt in an iron crucible until it loses two thirds of its weight. The dose is gr. v–x.]

THE LONDON MEDICAL GAZETTE.

No. XI. Dec. 4.—On the Results of Amputations. By J. A. Lawrie, M.D. Professor of Surgery in Anderson's University, Surgeon to the Glasgow Royal Infirmary, Glasgow Lock Hospital, &c.
[A valuable statistical paper.]

No. XII. Dec. 11.—On Congenital Opacity of the Cornea. By Mr. S. Crompton, of Manchester (with Engraving).
On the Detection of Albumen in the Urine. By G. O. Rees, M.D. &c
Observations on the Advantages presented by the Employment of a Stethoscope with a flexible Tube. By Golding Bird, M.D. A.M.
On a New Method of Cure for Shortsightedness. By Aug. Franz, M.D.
[Berthold's method, mentioned in our last Number.]
On Lateral Curvature of the Spine; and on Strabismus. By James Braid, Esq. M.R.C.S.E. of Manchester.
On the Division of the Muscles of the Back in a Case of Lateral Curvature of the Spine. By T. Laycock, Esq. York.
Dissection of a Club-Foot. By Philip B. Ayres, M.B. M.R.C.S. of Thane.

No. XIII. Dec. 18.—On the Colourless Globules in the Buffy Coat of the Blood. By Mr. Addison, of Great Malvern.
Case of Poisoning with Binoxalate of Potass. By John Jackson, Esq. M.R.C.S.

No. XIV. Dec. 25.—On the Treatment of certain Diseases of the Brain. By E. Copeman, Esq.
Cases of Cynanche Tonsillaris. By Joseph Bell, Esq. of Banhead.

No. XV. Jan. 1.—Case of Œdema of the Face and Tongue, Ulceration, &c. By James Syme, Esq. L.R.C.S. Edin. of Alloa.
On the Position of the Placenta. By Joseph Bell, Esq. of Banhead.
On Chyle and Lymph. By G. O. Rees, M.D. Member of the Royal College of Physicians of London, and Physician to the Northern Dispensary.

No. XVI. Jan. 8.—Note on the present Epidemic of Smallpox, and on the Necessity of Arresting its Ravages. By William Farr, Esq.
On the Treatment of Ulcers of the Lower Extremities. By J. Bell, Esq. of Barrhead.
On the Structure of Cartilage. By James Douglas, Esq. of Glasgow.
Case of severe Injury of the Head. By Edmund J. Furner, Esq. of Brighton.
Case of Dislocation of the Tibia inwards, with the Astragalus, and Fracture of the Fibula. By R. G. Coombs, Esq. of Newcastle, Staffordshire.

No. XVII. Jan. 15.—On Phlebitis of the Cerebral Sinuses as a Result of Purulent Otorrhœa. By James Bruce, M.D. Liverpool, formerly House-Surgeon to the Glasgow Royal Infirmary.
On the Coagulation of the Blood after Death. By J. Paget, Esq. M.R.C.S. Demonstrator of Morbid Anatomy, and Hon. Curator of the Museum, at St. Bartholomew's Hospital.
Case of Double Uvula. By George Bolster, Esq. of Tullerboy, Croom, Ireland.

No. XVIII. JAN. 22.—On Phlebitis of the Cerebral Sinuses as a Result of Purulent Otorrhœa. By J. Bruce, M.D. Liverpool.
Remarks on Strabismus, including an Analysis of Two Hundred Cases. By C. Radclyffe Hall, Esq. Manchester.
Description of a new Catheter. By J. C. Foulkes, Esq. Liverpool.
On a new Suspension Apparatus for Fractures of the Leg. By J. Lake, Esq.
Dissection of a Case after the Operation for Strabismus. By P. G. Hewett, Esq.
On the Agency of Sound in the Human Ear. By W. Shand, Esq. Glasgow.

No. XIX. JAN. 29.—On Pulmonary Emphysema. By R. H. Goolden, M.D. Fellow of the Royal College of Physicians, Physician to the Seamen's Hospital, Dreadnought. (With Engraving.)
An Account of some Cases of unnatural Narrowness of the Aorta, and of the Consequences of this Malformation: with Observations on the Causes which produce the Communications between the two sides of the Heart. By T. Wilkinson King, Esq. Lecturer on Pathology at Guy's Hospital.
On the Colourless Globules in the Buffy Coat of the Blood. By Wm. Addison, Esq. of Great Malvern (continued). (With Engravings.)
Case of Death from Carbonic Acid. By Charles Collambell, Esq.

No. XX. FEB. 5.—On Muscæ Volitantes. By A. L. Wigan, Esq. of Brighton.
Operation for the Cure of Wry-neck. By H. Symes, Esq. M.R.C.S.L. of Bridgewater.
On Hip Disease and Lumbar Abscess. By Wm. Oliver Chalk, Esq. Resident Surgeon to the R. S. B. Infirmary.
Arsenic in Sulphuric Acid. By G. O. Rees, Esq.

No. XXII. FEB. 19.—On the presence of Arsenic in Sulphuric Acid. By Henry Hough Watson, Esq. of Bolton-le-Moors.
King's College Hospital Report for 1840. By Wm. Augustus Guy, M.B. Cantab. Professor of Forensic Medicine, King's College, London; and Assistant-Physician to King's College Hospital.
Remarks on the Physiology of the Orbital Oblique Muscles, and on Strabismus. By E. Hocken, M.D. of Exeter.
On the Use of Secale Cornutum. By J. Hodgson, Esq.
Case of Rare Malformation of the Heart. By Albert Napper, Esq.
Some Remarks on Pneumonia in connexion with, or as a consequence of, Surgical Operations and Injuries. By J. E. Erischen, Esq. formerly House-Surgeon to University College Hospital.

No. XXIII. FEB. 26.—On Hip Disease and Lumbar Abscess. By Wm. Oliver Chalk, Esq. Resident Surgeon to the Royal Sea-Bathing Infirmary, Margate.
Case in which Death occurred after a Natural Labour. By John Chatto, Esq.
Case of extensive Abscess situated between the Crico-and Thyro-Arytenoidei Muscles and the lining Mucous Membrane. By Samuel B. Cowan, Esq. M.R.C.S. of Harrow.

---

### THE DUBLIN MEDICAL PRESS.

No. C. DEC. 2.—Successful Employment of the Gum-Elastic Tube in Tympanitis of Fever. By D. Donovan, M.D. of Skibbereen.

No. CI. DEC. 9.—On some Uses to which Sir James Murray's "Fluid Magnesia" may be advantageously applied. By M. Donovan, Esq.

No. CII. DEC. 16.—Case in which a Needle lodged in the Throat, and afterwards passed out through the Submaxillary Gland. By R. Maffett, M.D. Glasslough.

No. CIII. DEC. 23.—Division of the Muscles of the Back in a case of Lateral Curvature of the Spine. By T. Laycock, M.D. of York.
A case of Mammary Abscess treated by Compression. By Joseph Bell, Esq. of Glasgow.

No. CIV. DEC. 30.—Case of Amenorrhœa treated by Mustard Sinapisms. By A. G. Guinness, M.D. Clontarf.

No. CV. JAN. 6.—On Paralytic and other Nervous Diseases of the Eye. By Dr. Jacob.
On the Medicinal use of Acetate of Morphia. By M. Donovan, Esq.

No. CVI. JAN. 13.—On the use of Hydriodate of Potass in Typhus Fever. By G. T. Smyth, Esq. Kinlough Dispensary.
Case of Double Uvula. By G. Bolster, Esq. Tullerboy.
Treatment of Amenorrhœa by Sinapisms. By J. Mee, Esq. Dufanaghy.

No. CVII. Jan. 20.—Case of fatal Asphyxia from covering the face in bed. By R. Cranfield, M.B. Enniscorthy.

No. CXII. Feb.—On Diseases of the Genito-Urinary Organs in Childhood. By Chr. Fleming, Esq. M.D.
Some Observations on Hooping Cough. By Henry Kennedy, M.B. L.R.C.S.I.

---

### PROVINCIAL MEDICAL AND SURGICAL JOURNAL.

No. X. Dec. 5.—A fatal case of Diabetes, with Remarks. By Charles Hastings, M.D.

No. XI. Dec. 12.—Contributions to the Pathology of Children. By P. Hennis Green, M.D. (Scrofulous Tubercle of the Brain, continued in three Numbers.)

No. XII. Dec. 19.—Cases of Diabetes, with Remarks. By C. A. Bree, Esq. Stowmarket.
Dissection of two Cases of Rabies. By Mr. V. Pettigrew, M.D.

No. XIII. Dec. 26.—Case of Ischuria Renalis. By George Fife, M.D. Newcastle.

No. XIV. Jan. 2.—Case of Hydrophobia. By J. Toogood, Esq. Bridgewater.

No. XVII. Jan. 23.—Case of Polypus of the Uterus, and of Viper Bite. By J. Toogood, Esq.

No. XVIII. Jan. 30.—Case of Monstrosity. By S. N. Parsons, Esq. Wincanton.

No. XX. Feb. 13.—Case of Scirrhus of the Optic Thalamus and Corpus Striatum. By J. Waters, M.D.

---

### THE LONDON, EDINBURGH, AND DUBLIN PHILOSOPHICAL MAGAZINE.

No. CVII. Nov. 1840.—Observations on certain Peculiarities of Form in the Blood-corpuscles of the Mammiferous Animals. By S. Gulliver, Esq. F.R.S.

---

### *On the Structure of the Placenta.* By Dr. John Reid, F.R.C.P.E., Lecturer on Physiology, &c.

It is with great pleasure that we find ourselves again called upon to notice the results of Dr. Reid's industry and skill in the prosecution of anatomico-physiological enquiries; and our pleasure is the greater in the present instance, because his recent labours have given what is to our minds a very satisfactory solution of a long-disputed question of no slight interest, the connexion between the fœtus and the uterus of the human female. We shall briefly state the facts observed by Dr. Reid, and his inferences from them.

In dissecting the uterus, with the attached placenta, of a woman who had died suddenly during the seventh month of pregnancy, Dr. Reid's attention was attracted towards a number of rounded bands, passing between the uterine surface of the placenta, and the inner surface of the uterus. These bands [which were distinct from the utero-placental vessels described by the Hunters] were generally observed to become elongated, thinner, and of a cellular appearance when put on the stretch, and were easily torn across; whilst at other times, though much more rarely, *they could be drawn out in the form of tufts from the mouths of the uterine sinuses.* Some of these tufts were found to be much more elongated than others, occasionally extending an inch from the open mouth of the sinus by which they had entered, and even passing into a neighbouring sinus,—and sometimes scarcely dipping into the sinus. These tufts were clearly ascertained to belong to the fœtal system, being filled by injection of the umbilical vessels; and it was further ascertained that each consisted of ramifications of the umbilical veins and arteries, bound together so as to form apparently but one set of filaments,—a structure exactly corresponding, therefore, (as Dr. Reid justly remarks,) with the branchial filaments of aquatic animals. These tufts lay within the uterine sinuses in some degree loose, but were observed to be bound down at various points by reflexions of the inner coat of the venous system of the mother upon their outer surface (just as, it may be pointed out, the intestines are supported in the abdominal cavity, by the reflexions of the peritoneum.) It is on account of this kind of union, that the tufts cannot readily be withdrawn from the sinuses, the connecting vessel usually giving way in preference.

With this clue Dr. Reid proceeded to examine minutely the structure of the placenta itself; and he succeeded in demonstrating that the greater part of its substance exactly corresponds with the tufts just described; being composed of bundles of filaments interwoven with each other, each filament being composed, like those of the tufts, of a ramification of the umbilical artery and vein. There is no cellular or any other tissue filling up the spaces between the branches of the fœtal placental vessels; the so-called cells being merely the interstices between the interlacing tufts, which tufts do not seem, in spite of their close approximation, to be actually connected with each other. Upon tracing the coats of some of the utero-placental veins, which contained none of the tufts, and of the curling arteries, through the decidua it was found that they were reflected over these prolongations of the umbilical vessels within the placenta; just as the lining of the uterine sinuses is reflected over the tufts which projects into them. The interior of the placenta is thus shown to consist of the ramifications of the umbilical vessels, the blunt terminations of which are ensheathed in prolongations of the inner coat of the vascular system of the mother, or at least in a membrane continuous with it.

If we adopt this view of the structure of the placenta, the blood of the mother when it passes into that organ, through the curling arteries of the uterus, may be regarded as entering a large sac formed by the inner coat of the vascular system of the mother, and this is intersected in many thousands of different directions, by the placental tufts prosecting into it like fringes, and pushing its thin wall before them in the form of sheaths, which thus closely envelope both the trunk and each individual branch composing these tufts. Round this sac the maternal blood is returned by the utero-placental veins, without having been extravasated, or without having left her own system of vessels. Into its cavity, the tufts of the placenta hang, like the branchial vessels of aquatic animals, and are bathed in the blood of the mother, just as are those tufts which project into the sinuses. This sac is protected and strengthened on the fœtal surface of the placenta by the chorion; on the uterine surface by the decidua vera (of the existence of which in this position Dr. Reid has satisfied himself), and on the edges or margin by the decidua reflexa. The blood of the mother and that of the fœtus can readily act on one another through the spongy and cellular walls of the placental vessels and the thin sac ensheathing them; just as the blood in the branchial vessels of aquatic animals is acted on by the water in which they float. According to this view of the structure of the placenta (which, when once understood, is found to be characterized by extreme simplicity, as well as by conformity to analogy), there is no proper division into fœtal and maternal portions, since tufts of placental vessels with their blunt terminations may be found immediately under the chorion covering its fœtal surface, as well as towards the uterine surface.

From a small number of observations made upon uteri and placentæ of different ages, Dr. Reid is disposed to conclude (though he by no means wishes to state it as a positive fact) that the degree in which the tufts of fœtal vessels project into the uterine sinuses increases with the advance of gestation. When the placenta is detached, at the conclusion of delivery, it would seem that most if not all of the tufts are retained within the uterine sinuses, being kept there by the connexions already mentioned. We think it not unlikely, however, that some may be occasionally drawn out, but escape observation; and we would suggest to those of our readers whose practice affords them facilities for so doing, that they take pains to ascertain this fact, by immersing the placenta in water, as soon as may be after its separation, and carefully observing whether any such tufts hang down from the placental surface, when it is placed horizontally and on looking downwards. Whether this be the case or not, however, the existence of the tufts has been placed beyond a doubt by Dr. Reid, who has satisfied Professors Alison, Allen Thomson, and J. Y. Simpson of their character; and Dr. Sharpey has given the weight of his testimony, based upon independent observation. Feeling, as we do, great confidence in Dr. Reid's caution and sagacity as well as anatomical skill, we have little hesitation in according with the general view of the structure of the placenta, by which he has been led by this interesting discovery; and we trust that it will soon receive full confirmation from the researches of others.

Dr. Reid's paper contains, besides an account of his own researches, a summary of the opinions of others on this *questio vexata.* Of these, Weber's views appear to present the nearest approach to his own. Weber had not noticed any prolongation of the fœtal vessels beyond the placenta; but he had spoken of the continuation of the venous canals of the mother into the placenta, where, according to him, they dilate into large sinuses, upon the walls of which the placental tufts are not only ramified, but also project into the interior, carrying the walls of the sinuses before them, much in the manner described by Dr. Reid. The chief difference between the two sets of views consists therefore in this,—that Dr. Reid considers the interior of the placenta in the light of one large sac, continuous with the uterine vessels, having its cavity divided by the interlacement of the ramifications of the fœtal vessels, which are covered by the reflexions of the lining of the sac, into numerous small cells communicating with each other, whilst, according to Weber, several smaller cavities or sinuses are contained in the substance of that organ.

Those who are curious in Morphological Science will not have much difficulty in perceiving a strong analogy between the mode in which the fœtal blood is thus renewed, by being brought into relation with the maternal fluid, and that in which the blood is aerated in the mollusca. The relative positions of the placenta and of the branchiæ (the structure of which has been shown to be essentially the same) in respect to the heart, rectum, &c., have a very close correspondence.

*Edinb. Med. and Surg. Journal. Jan.* 1841.

---

### *On some Points in the Anatomy of the Medulla Oblongata.*
By John Reid, m.d. f.r.c.p.e., &c.

The object of this short paper is to correct an error which prevails in all the anatomical works of this country, in reference to the connexion between the spinal cord and the medulla oblongata, which has given rise to much theoretical difficulty, in regard to the origins of some of the nerves. The error is in the idea that the decussating fibres of the anterior pyramids pass down the anterior columns of the spinal cord; the fact being that they pass down the middle or lateral columns. If the course of the pyramidal fibres be traced to their decussation, it will be found that those which cross each other pass backwards as well as downwards, so as to unite with the fibres continuous with the restiform bodies in forming the lateral or middle columns; and Dr. Reid states it as the result of repeated examination, that *none of the decussating fibres pass down the anterior columns.* In this assertion he is supported in part by Rosenthal, Cruveilhier, and Arnold, who all speak of the pyramids as principally derived from the middle columns. But a portion of the pyramidal fibres (the amount of which seems to vary much in different cases) does not decussate; and these unite with those of the olivary bodies, to form the anterior column of the cord. Besides these, there is the *arciform* band of non-decussating fibres, which goes backwards to join the posterior column as it passes into the crus cerebelli.

The decussation of the fibres of the middle column may be examined from behind, by detaching the posterior columns, and has been described as such by Sir C. Bell; but he does not state that the decussating fibres pass into the pyramidal column of the opposite sides, and not into the middle columns.

The olivary bodies are separated from each other at the upper part of the medulla oblongata by the pyramids; but when the main body of the fibres of the latter pass backwards, the olivary columns approach each other, so as to be only separated by the anterior median fissure. On tracing the fibres of the olivary column upwards, they are said by Dr. Reid to inclose the olivary body like a kernel, and then to pass into the pons varolii, where they divide into two bands, of which one proceeds upwards and forwards to join the crus cerebri, whilst the other proceeds upwards and backwards to the corpora quadrigemina.

The olivary columns afford attachment to the anterior roots of the first and second cervical, and of all the other spinal nerves; they may therefore be regarded, without much hesitation, as forming a motor tract. On tracing it upwards, it is found to give origin to the *portio dura*; the *hypoglossal* and *abducens* in part arise from it, and in part from the pyramidal column; the *small root of the fifth*, and the *trochlearis* arise from that portion of it which runs to the corpora quadrigemina. The *large root of the fifth* is traced by Dr. Reid into the restiform or middle column; from which also arise all the fibres of the *glosso-pharyngeal*, and nearly all those of the *par vagum* (which are not connected with the olivary bodies, as Mr. Solly supposed); a few of these last being usually seen to arise from the arciform band of the pyramidal column, when this is well marked. The *spinal accessory* nerve is believed by Dr. Reid to originate from the decussating fibres of the pyramidal columns; but on this point he does not speak with certainty.

Of the function of the decussating fibres, Dr. Reid thinks we have at present no sufficient means of forming an opinion. Their connexion with the roots of the spinal nerves is by no means evident; and their position throws great difficulty in the way of experiment. Dr. Reid observed that in two kittens deprived of sensation and voluntary motion by prussic acid, extensive muscular movements were produced by irritating the pyramidal bodies with the point of a needle; but whether the impression thus produced was conveyed directly to the muscles or by a reflex action, he could not satisfy himself.

*Edinburgh Medical and Surgical Journal. Jan.* 1841.

---

*On Bleeding in Mania.* By W. A. F. BROWNE, M.D. Superintendent of the Dumfries Asylum.

DR. BROWNE not only condemns the use of bloodletting in mania, but goes so far as to say that "in nine cases out of ten, when a professional man even of eminence is called to a case of recent mania, he orders general depletion, the liberal exhibition of the solution of tartar emetic, brisk cathartics, and cold applications to a shaven scalp. If the symptoms, and especially the violent and rapid pulse, continue or return in unabated force, the patient is perhaps bled again from the arm, or if not, he will be cupped and leeched to a certainty. And after these energetic measures are manfully urged for days or weeks, and the tartar-emetic lustration fails in every respect, except in producing a nausea quiet, an asylum is recommended as the last resource." Dr. Browne then states that such treatment is not confined to mania: "The melancholic monomaniac and maniac, and all the species included under these general terms, are its objects or its victims. The subject is too momentous for ridicule; but ridiculous and indefensible must that system be which includes all the foregoing diseases under one category, and draws blood and drenches upon the same principle and with the same degree of discrimination as a leech."

We agree entirely with Dr. Browne in thinking the subject too momentous for ridicule, but we consider it as at the same time too momentous for exaggeration; and we cannot help fearing that in his anxiety to warn his readers against the injury arising from the indiscriminate use of the lancet in mental affections, Dr. Browne has unconsciously and unjustifiably allowed himself to bring a strong general charge against his brethren, which, if true to the extent which he alleges, would be a reproach and disgrace to them. We believe that in many cases bleeding and tartar emetic *are* used where they had been better omitted. But so far as our experience and information go, we have no hesitation in affirming that instead of nine out of ten intelligent general practitioners following the culpable course here ascribed to them, those who do so constitute only a small minority of the whole. From the strong language used by Dr. Browne, and his charge being specially extended to the intelligent and eminent among general practitioners, we are forced to the conclusion that he must have either overstated his case, or been unusually unfortunate in meeting with so many instances of mismanagement. We concur cordially with him in his objections to general bleeding and nauseating in mania, in his anxiety for the early removal of the insane to an asylum, in the general propriety of a generous diet, and we are as earnest as he can be that no cases of maltreatment should ever occur. But for that very reason we contend that he ought either greatly to have restricted his charge against his professional brethren, or *to have produced proofs to substantiate it.* We know that cases such as he describes are to be met with more frequently than is creditable to the parties concerned, but after twenty years' experience we can honestly aver that instead of their amounting to nine cases out of ten, of those treated by well-informed and sensible practitioners, as Dr. Browne assumes, they have been few and far between compared with those treated without general depletion. And on enquiry among our medical acquaintance, we should say that instead of nine out of ten practitioners, "even of eminence," falling into the error imputed to them, the large majority would condemn it as heartily as Dr. Browne himself. Appearing, as this statement now does, *without proofs* it is calculated to rouse in the minds of Dr. Browne's brethren an unfriendly feeling towards himself, and thereby to diminish his influence when really in advance of them, by diminishing their confidence in his accuracy and judgment.

*Edinburgh Monthly Journal. Feb.*, 1840.

---

### *On the Advantages of a Stethoscope with a Flexible Tube.*

By Golding Bird, M.D. A.M.

The end which is applied to the chest consists of a thin cup of ebony, an inch in diameter, and carefully rounded at its edges: a flexible tube, from sixteen to twenty inches in length, formed of a spiral iron wire, covered with caoutchouc, and bound tightly round with silk or velvet, having an internal diameter of about one fourth of an inch, is fixed into the apex of this cup: the other end of the tube is cemented into a perforated ebony ball, on the top of which is screwed a slightly concave plate of ivory, two inches in diameter, also perforated in its centre. When this instrument is used, the ebony cup should be held, between the fingers and thumb of one hand, against the walls of the chest, and thus, with the utmost facility, and without producing unnecessary pressure, a tolerably air-tight approximation is effected. The ebony ball at the other end should be held in the other hand, and the ivory plate closely applied to the ear. In this manner we have a column of air, bounded at one extremity by the vibrating surface of the chest, and at the other by the membrana tympani, which thus becomes placed in the position most favorable for assuming vibrations throughout its whole extent. The cup of ebony has its vibrations *damped* by the pressure of the fingers grasping it, whilst the covering of silk or velvet performs a similar office, with sufficient accuracy, for the spiral wire forming the tube of the instrument, whilst from the convenient position in which the physician can place himself with regard to the patient, the necessarily close approximation of the end of the stethoscope to the surface of the chest may be effected. In addition to this, the intensity of sound does not appear to be sensibly diminished by the flexure of the tube, and thus we can auscultate the sides and back of the chest by a very slight movement of the body of a patient whilst in the recumbent position.

*London Medical Gazette. Dec.* 11, 1840.

